# Incidence and risk factors of post-operative cognitive decline after ablation for atrial fibrillation

**DOI:** 10.1186/s12872-021-02139-7

**Published:** 2021-07-14

**Authors:** Jing Zhang, Shi-Jun Xia, Xin Du, Chao Jiang, Yi-Wei Lai, Yu-Feng Wang, Zhao-Xu Jia, Liu He, Ri-Bo Tang, Jian-Zeng Dong, Chang-Sheng Ma

**Affiliations:** 1 Department of Cardiology, Beijing Anzhen Hospital, Capital Medical University, National Clinical Research Center for Cardiovascular Diseases, No. 2 Anzhen Road, Chaoyang District, Beijing, 100029 China; 2 Department of Cardiology, Affiliated Hospital of Hebei University, Baoding, 071000 China

## Abstract

**Background:**

Catheter ablation is widely used in atrial fibrillation (AF) management. In this study, we are aimed to investigate the incidence of postprocedural cognitive decline in a larger population undergoing AF ablation under local anesthesia, and to evaluate the associated risk factors.

**Methods:**

This study included 287 patients with normal cognitive functions, with 190 ablated AF patients (study group) and 97 AF patients who are awaiting ablation (practice group). We assessed the neuropsychological function of each patient for twice (study group: 24 h prior to ablation and 48 h post ablation; practice group: on the day of inclusion and 72 h later but before ablation). The reliable change index was used to analyze the neuropsychological testing scores and to identify postoperative cognitive dysfunction (POCD) at 48 h post procedure. Patients in the study group accepting a 6-month follow up were given an extra cognitive assessment.

**Results:**

Among the ablated AF patients, 13.7% (26/190) had POCD at 48 h after the ablation procedure. Multivariable analysis revealed that, a minimum intraoperative activated clotting time (ACT) < 300 s (OR 3.82, 95% CI 1.48–9.96, *P* = 0.006) and not taking oral anticoagulants within one month prior to ablation(OR 10.35, 95% CI 3.54–30.27, *P* < 0.001) were significantly related to POCD at 48 h post-ablation. In 172 patients of the study group accepting a 6-month follow up, there were 23 patients with POCD at 48 h post-ablation and 149 patients without POCD. The global cognitive scores were decreased in 48 h post-operation tests (0 ± 1 vs − 0.15 ± 1.10, *P* < 0.001) and improved significantly at 6 months post-operation (0 ± 1 vs 0.43 ± 0.92, *P* < 0.001). In the 23 patients with POCD at 48 h after the procedure, global cognitive performance at 6 months was not significantly different compared with that at baseline (− 0.05 ± 1.25 vs − 0.19 ± 1.33, *P* = 0.32), while 13 of them had higher scores than baseline level.

**Conclusions:**

Incident of POCD after ablation procedures is high in the short term. Inadequate periprocedural anticoagulation are possible risk factors. However, most POCD are reversible at 6 months, and a general improvement was observed in cognitive function at 6 months after ablation.

## Introduction

Atrial fibrillation (AF) ablation has been adopted as a first-line therapy in symptom control and the improvement of patients' quality of life (QoL) [1–3]. However, subclinical cerebral ischemia is common during AF ablation [4], which may result in postprocedural cognitive impairment. Studies on the association between AF ablation and cognitive impairment are limited. The incidence of postoperative cognitive dysfunction (POCD) is reported to be as high as 28% in 48 h after AF ablation, and it seems part of them are reversible [5]. However, current studies are inconsistent in terms of the association between AF ablation and POCD [6–9]. Additionally, the ablation procedures in most current studies were carried out under general anesthesia. Whether the POCD is associated with general anesthesia or AF ablation per se, and if associations exist with other factors were still unknown.

In this study, we aim to describe the incidence of postprocedural cognitive decline in a larger population undergoing AF ablation under local anesthesia, and to evaluate the associated risk factors.

## Methods

### Study population

From March 2017 to August 2017, AF patients in the AF ablation waiting list of Beijing Anzhen Hospital were enrolled for primary cognitive screening. Patients over 60 years of age with normal cognitive functions were included and further randomly divided into ablated AF patients (study group) and AF patients who are awaiting ablation (practice group). Exclusion criteria included pre-existing neurological or clinically evident neurovascular disease, significant pre-morbid depression and/or anxiety, anticipated difficulty with neurocognitive assessment (e.g., deafness, language difficulties), and with history of cardiopulmonary bypass surgery.

In the study group, cognitive assessment was administered at 24 h prior to ablation and 48 h after the ablation procedure respectively. The practice group was set up to determine the practice effect of cognitive tests resulted from repeated testing, two consecutive cognitive assessment were administered to them with a 72-h interval before the ablation. All patients provided a written informed consent for both the baseline and post-procedural cognitive assessments.

In patients of the study group consenting to accept an extra 6-month cognitive assessment at enrollment, practice effect was not considered in 6-month cognitive assessment, for previous study have reported similar test results between the baseline and 3-month assessment in non-ablated AF patients [10].

### Neuropsychological testing

A trained interviewer administered neuropsychological testing to all included patients. A well-established formal testing battery consisting of nine tests were used, which is based on the Canadian Study of Health and Aging [11–15] (Appendix Table 4).

The interviewer recorded both the number of correct answers and the time taken to complete the tests. To reduce the potential influence by other factors, parallel neuropsychological testing was conducted in the same room, by the same interviewer, and at the same time of the day for each recruited patient.

Visual analog scales were also utilized to assess the presence of mood disorder levels which may influence the scores on neuropsychological tests. Patients were asked to estimate their current levels of depression and anxiety by marking a line standardized to 10 cm in length [13].

### Definition of POCD at 48 h after ablation

The reliable change index (RCI) was used to analyze the neuropsychological testing scores and to identify POCD [15, 16].The details of the RCI calculation are listed as follows:

For all the patients in both the study group and the practice group, the initial testing raw score (X1) was subtracted from the final testing raw score (X2) to produce a ΔX for each task except the timed tasks. We subtracted the final testing raw score (X2) from the initial testing raw score (X1) to calculate a ΔX for the timed tasks (Trail Making Task, Parts A and B, and Grooved Pegboard Test, Dominant and Non-dominant Hands).

To eliminate any learning and practice effects, we subtracted the mean difference [Mean(ΔXp)] of the practice group from the change in study group patient's testing scores (ΔX) and then divided it by the SD of the change in test results of the practice group [SD(ΔXp)], controlling for the expected variability, to obtain the final z-score for every test.

- Z-score = [(X2-X1)-Mean(ΔXp)]/SD(ΔXp) (for every test except the timed tasks)
- Z-score = [(X1-X2)-Mean(ΔXp)]/SD(ΔXp) (for the timed tasks)

A combined test score (∑Z_combined_) was created by using the sum of z-scores for each test (**∑**Z 1,2,3…9) divided by the SD of this summation in the practice group (SD [∑Z_practice_ 1,2,3…9]).

$$\sum Z_{{{\text{combined}}}} = \sum Z{\text{ }}1,2,3 \ldots 9/SD{\text{ }}(\sum Z_{{{\text{practice}}}} 1,2,3 \ldots 9)$$

We defined the presence of POCD when the z-score < − 1.96 on ≥ 2 tests, or the combined z-score < − 1.96 [15, 16].

### Cognitive assessment in 6-month follow-up

Comparisons of neuropsychological outcomes were made among pre-operation, and 48 h and 6 months post-operation. The mean difference between cognitive assessments in the practice group was defined as practice effect and was subtracted from raw scores of 48 h postoperative tests in the study group. All test results were transformed to z scores. A global cognitive score was created by averaging the z scores of each subtest and standardizing them [17]. Absolute test scores were reversed for timed tasks, hence higher z scores always represent better test performance.

### Perioperative anticoagulation management

Transesophageal echocardiography (TEE) was performed in every patient to rule out the left atrium or atrial appendage thrombus before the AF ablation procedure.

During admission, for patients on warfarin, a nonstop strategy was used (i.e., no low-molecular-weight heparin LMWH bridging) if the international normalized ratio (INR) value was between 2.0 and 3.0. If the patient's INR value was below 2.0, bridging with LMWH while titrating warfarin was conducted until a therapeutic INR was obtained. If the INR value was above 3.0, we titrated the warfarin dose by stopping or reducing the warfarin dose under close monitoring. For patients on new oral anticoagulants (NOAC), we stopped using NOAC after admission and bridged with full-dose LMWH and reinitiated NOAC at six hours after the ablation procedure. For those not on warfarin or NOAC at admission, full-dose LMWH was administered, and post-procedure oral anticoagulation use was a shared decision between doctors and patients. All patients were treated with anticoagulants for at least 3 months postoperatively, and then at 3 months, patients with a CHA2DS2-VAS score >  = 2 were encouraged to continue using oral anticoagulation.

During the procedure, ACT was measured every 30 min to maintain an ACT level above 300 s.

### AF ablation procedure

After overnight fasting, the patients received AF ablation procedures under conscious sedation with fentanyl. Circumferential pulmonary vein isolation was applied for the patients with paroxysmal atrial fibrillation (PAF) [18, 19] while a fixed '2C3L' approach for patients with non-paroxysmal AF [20] were applied, guided by a three-dimensional electroanatomic mapping system (CARTO, Biosense-Webster, Inc.). The '2C3L' strategy is a fixed approach for ablation of persistent AF, which consists of bilateral circumferential pulmonary vein antrum isolation ('2C') and three linear ablation sets ('3L').Linear ablation is empirically applied across the mitral isthmus, the left atrial roof, and the cavo-tricuspid isthmus. Ablation energy was delivered with a maximum power of 30 to 35 W with continuous heparinized saline flow at a rate of 17 mL/min.

### Statistical analysis

The data were reported as the mean ± standard deviation (SD) for continuous variables and frequency (%) for categorical variables, while the Student’s t-test and chi-squared test were deployed to assess the differences between the groups. Univariate logistic regression analysis was performed to assess the association of each variable with POCD. The variables with a *P* value < 0.05 in the univariate analysis and of clinical importance were then included in the multivariate logistic regression analysis, including the CHA_2_DS_2_-VASc score (< 2 for males, < 3 for females), the minimum intraoperative ACT (< 300 s), and non-administration of oral anticoagulants within the one month prior to the ablation. Probability values less than 0.05 were considered as significant difference. All analyses were conducted using SPSS version 22.0 for Windows.

## Results

### Baseline characteristics

From March 2017 to August 2017, 625 AF patients in the AF ablation waiting list of Beijing Anzhen Hospital were enrolled for primary cognitive screening. After excluding 238 patients under 60 years of age, 29 patients with a history of stroke, 2 patients who suffered from severe neurological/psychiatric disorders, 6 patients with a history of depression/anxiety, 7 patients who suffered from anticipated difficulty in neurocognitive assessment (e.g., deafness, language difficulties), 4 patients who took surgeries under cardiopulmonary bypass grafting, and another 39 patients with cognitive impairment assessed by the Mini-Mental State Examination (MMSE) test prior to the ablation procedure, 300 cognitively normal patients were included in the study.

**Fig. 1:**
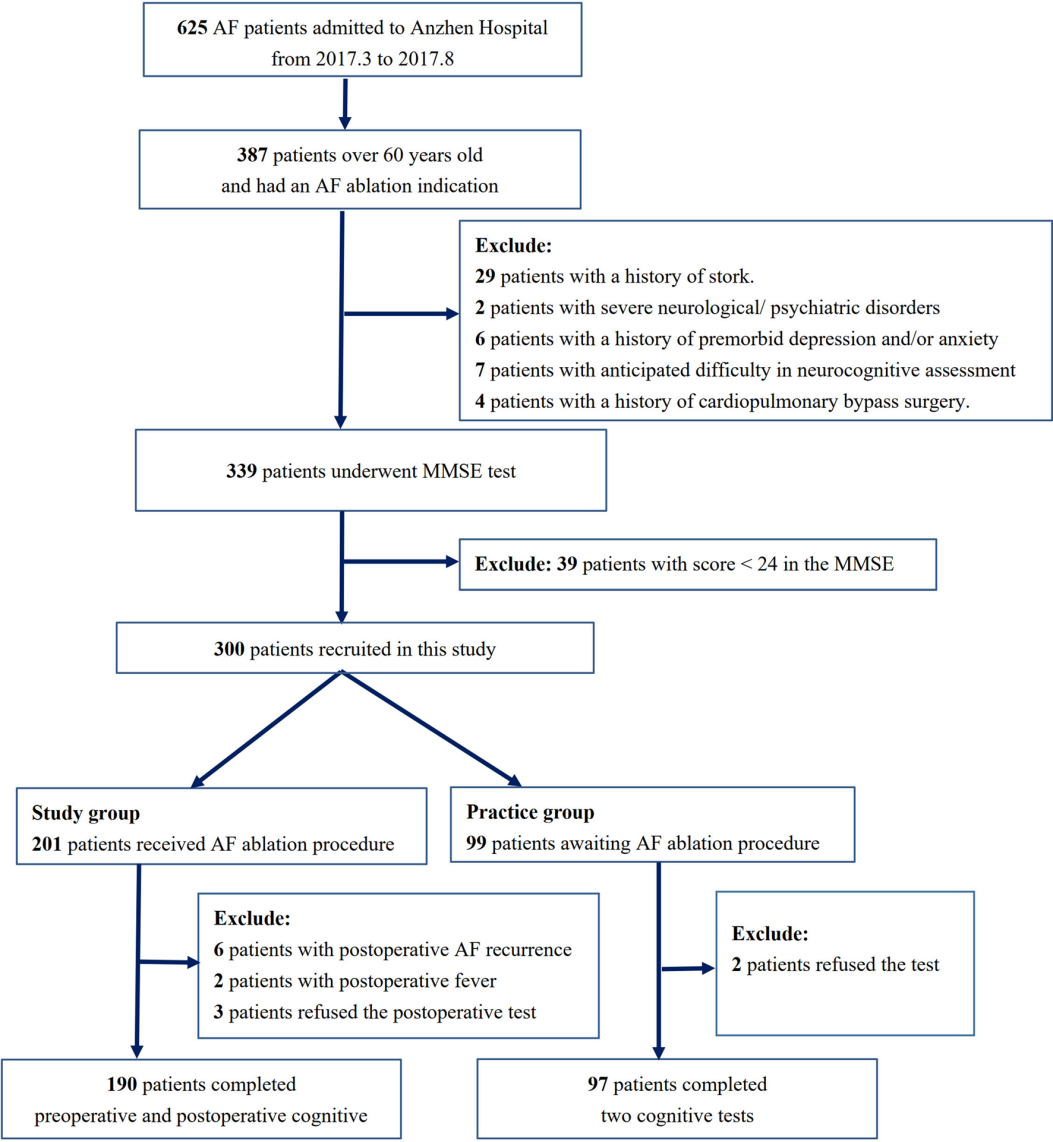
Flowchart of the study

Among them, 99 patients were randomly assigned to the practice group to determine the practice effect of cognitive tests resulted from repeated testing, two consecutive cognitive assessment were administered to them with a 72-h interval before the ablation. For 201 patients assigned to the study group, cognitive assessment was administered at 24 h prior to ablation and 48 h after the ablation procedure respectively. Thirteen patients did not complete the postoperative tests because of postoperative AF recurrence (6 patients), postoperative fever (2 patients), and withdrawal of consent (5 patients). Finally, data from 190 patients in the study group and 97 patients in the practice group were included in the current analysis (Fig. 1).

The characteristics of 190 patients in the study group and 97 patients in the practice group were listed in Table 1. The demographic characteristics of the two groups were similar with respect to male gender (59.5% vs 54.6%, *P* = 0.43), age (66.6 ± 5.4 vs 67.5 ± 5.0, *P* = 0.11), high educational level (30% vs 32%, *P* = 0.73), and PAF (68.9% vs 64.9%, *P* = 0.49). No significant differences in the prevalence of comorbidities and current medication were observed between the two groups.

**Table 1:** Baseline characteristics

Variable	Study group (n = 190)	Practice group (n = 97)	*P* value
Age (years)	66.6 ± 5.4	67.7 ± 5.0	0.11
Male	113 (59.5)	53 (54.6)	0.43
College and above	57 (30.0)	31 (32.0)	0.73
Paroxysmal atrial fibrillation	131 (68.9)	63 (64.9)	0.49
Diabetes	33 (17.4)	15 (15.5)	0.68
Hypertension	122 (64.2)	57 (58.8)	0.37
Dyslipidemia	41 (21.6)	25 (25.8)	0.42
Coronary heart disease	30 (15.8)	13 (13.4)	0.59
Heart failure	10 (5.3)	4 (4.1)	0.78
Level of anxiety#	3.8 ± 1.3	3.6 ± 0.7	0.14
Level of depression#	2.7 ± 1.3	2.7 ± 1.1	0.72
Smoker	33 (17.4)	14 (14.4)	0.52
CHA_2_DS_2_-VASc score (< 2 for males, < 3 for females)	89 (46.8)	40 (41.2)	0.38
Oral anticoagulants	116 (61.1)	56 (57.7)	0.59
Antiplatelet	19 (10.0)	12 (12.4)	0.54
ACEI/ARB	57 (30.0)	34 (35.1)	0.38
Statins	91 (47.9)	40 (41.2)	0.28

Values are presented as the mean ± SD or n (%)

^#^Level of anxiety and depression assessed by visual analog scales

### Perioperative anticoagulant strategies and procedural characteristics

In this study, all the ablated AF patients received TEE in admission. No left atrium or left appendage thrombi were found.

A total of 116 ablated patients were on oral anticoagulants for at least one month before the procedure. Among them, 27 patients who took warfarin orally before admission were treated with a nonstop strategy while 89 patients who took NOACs before admission were given LMWH bridging. All the other 74 ablated patients not on anticoagulants within one month before the procedure were given LMWH bridging.

Successful pulmonary vein isolation was achieved in all ablated patients. Sixty-two patients (32.6%) underwent adjuvant linear ablation. None of the patient developed a transient ischemic attack/stroke or had other perioperative clinical embolic events.

### Incidence of POCD at 48 h after ablation

Among the ablated patients, 26 out of 190 patients (13.7%) satisfied the definition of POCD at 48 h after ablation. Cognitive impairment was detected across the entire range of tests. The tests with the most prominent decrease in scores were the CERAD-I and CERAD-D (memory), the Trail Making Test A and B (executive functioning), and combined score (Z). The incidence of cognitive deficits (z-score was less than − 1.96) were significantly higher in the study group in CERAD-I (15.8% vs 3.1%, *P* = 0.001), CERAD-D (13.2% vs 4.1%, *P* = 0.02), Trail-A (11.4% vs 2.1%, *P* = 0.01), Trail-B (8.9% vs 2.1%, *P* = 0.03), and combined z score (10.0% vs 2.1%, *P* = 0.02) (Table 2). The global cognitive scores at baseline and 48 h post-ablation for the study group and the practice group were shown in Appendix Fig. 4.

**Table 2:** Impaired performance on neuropsychological tests in study group and practice group at 48 h after ablation

Variable	Study group (n = 190)	Practice group (n = 97)	*P* value
CERAD-I	30 (15.8)	3 (3.1)	0.001*
CERAD-D	25 (13.2)	4 (4.1)	0.02*
Trail A (s)	22 (11.4)	2 (2.1)	0.01*
Trail B (s)	17 (8.9)	2 (2.1)	0.03*
Digit symbol substitution	6 (3.2)	1 (1.0)	0.43
Controlled oral word association	8 (4.2)	3 (3.1)	0.76
CERAD Semantic Fluency	6 (3.2)	4 (4.1)	0.74
Grooved Pegboard Test-D (s)	6 (3.2)	0 (0)	0.10
Grooved Pegboard Tes-N (s)	6 (3.2)	0 (0)	0.10
Combined Score (Z)	19 (10.0)	2 (2.1)	0.02*

Values are presented as n (%)

The asterisk indicates statistical significance

### Factors associated with POCD at 48 h after ablation

The variables included in the univariate logistic regression were age, sex, education, AF type, comorbidities (i.e., diabetes, hypertension, dyslipidemia, coronary artery disease, heart failure), smoking status, medication use (i.e., oral antiplatelet drugs, ACEI/ARB, statins), CHA_2_DS_2_-VASc score, levels of depression and anxiety, and procedure-related factors (Table 3). The minimum intraoperative ACT of < 300 s and not taking an oral anticoagulant within one month before the ablation procedure were found to be significantly associated with POCD occurrence, which were then included in the multivariate logistic regression analysis.

**Table 3:** Univariable and multivariable adjustment for factors associated with POCD at 48 h after ablation in the study group

Variable	Patients with POCD (n = 26)	Patients without POCD (n = 164)	Univariable adjustment			Multivariable adjustment		
			OR	95% CI	*P* value	OR	95% CI	*P* value
Age (years)	67.7 ± 6.3	66.4 ± 5.3	1.04	0.97–1.12	0.26	–	–	–
Male	12 (46.2)	101 (61.6)	1.87	0.81–4.30	0.14	–	–	–
College and above	5 (19.2)	52 (31.7)	0.51	0.18–1.44	0.20	–	–	–
Paroxysmal atrial fibrillation	17 (65.4)	114 (69.5)	1.21	0.50–2.89	0.67	–	–	–
Diabetes	3 (11.5)	30 (18.3)	0.58	0.16–2.07	0.40	–	–	–
Hypertension	16 (61.5)	106 (64.6)	0.88	0.37–2.05	0.76	–	–	–
Dyslipidemia	4 (15.4)	37 (22.6)	0.62	0.20–1.93	0.41	–	–	–
Coronary artery disease	6(23.1)	24 (14.6)	1.75	0.64–4.80	0.28	–	–	–
Heart failure	3 (11.5)	7 (4.3)	2.96	0.71–12.12	0.14	–	–	–
Smoker	4 (15.4)	29 (17.7)	0.85	0.27–2.64	0.77	–	–	–
Oral antiplatelet drugs	4 (15.4)	15 (9.1)	1.81	0.55–5.94	0.33	–	–	–
ACEI/ARB	10 (38.5)	47 (28.7)	1.56	0.66–3.68	0.31	–	–	–
Statins	13 (50.0)	78 (47.6)	1.10	0.48–2.52	0.82	–	–	–
CHA_2_DS_2_-VASc score (< 2 for males, < 3 for females)	11 (42.3)	78 (47.6)	0.81	0.35–1.87	0.62	0.95	0.38–2.39	0.91
Level of depression	2.6 ± 1.4	2.7 ± 1.2	0.93	0.66–1.30	0.67	–	–	–
Level of anxiety	3.7 ± 1.6	3.8 ± 1.3	0.97	0.70–1.34	0.85	–	–	–
Intraoperative hypotension	0 (0.0)	2 (1.2)	0.00	0.00	1.00	–	–	–
Direct current cardioversion	7 (26.9)	34 (20.7)	1.41	0.55–3.63	0.48	–	–	–
Minimum intraoperative ACT < 300 s	12 (46.2)	38 (23.2)	2.84	1.21–6.66	0.02*	3.82	1.48–9.96	0.006*
Left atrial access time (min)	125.6 ± 32.6	120.2 ± 38.1	1.00	0.99–1.01	0.50	–	–	–
Radiofrequency duration (min)	83.1 ± 31.0	80.0 ± 34.1	1.00	0.99–1.02	0.66	–	–	–
Fentanyl dosage (μg)	161.3 ± 45.3	160.5 ± 48.4	1.00	0.99–1.01	0.93	–	–	–
Not on oral anticoagulant within 1-month prior to ablation	21 (80.8)	53 (32.3)	8.80	3.14–24.6	< 0.001*	10.35	3.54–30.27	< 0.001*
No oral anticoagulants interruption	3 (11.5)	24 (14.6)	0.76	0.21–2.73	0.68	–	–	–

Values are presented as the mean ± SD or n (%)

OR, odds ratio; CI, confidence interval; the asterisk indicates statistical significance

In the multivariate adjustment, the minimum intraoperative ACT < 300 s (OR 3.82, 95% CI 1.48–9.96; *P* = 0.006) and not taking oral anticoagulant within one month before the ablation procedure (OR 10.35, 95% CI 3.54–30.27; *P* < 0.001) were significantly associated with POCD occurrence, while CHA_2_DS_2_-VASc (< 2 for males, < 3 for females) was not related to POCD (OR 0.95, 95% CI 0.38–2.39, *P* = 0.91). The univariable and multivariable adjustment for factors associated with POCD at 48 h after ablation in the study group were shown in Table 3.

### Cognitive outcomes in 6-month follow-up

**Fig. 2:**
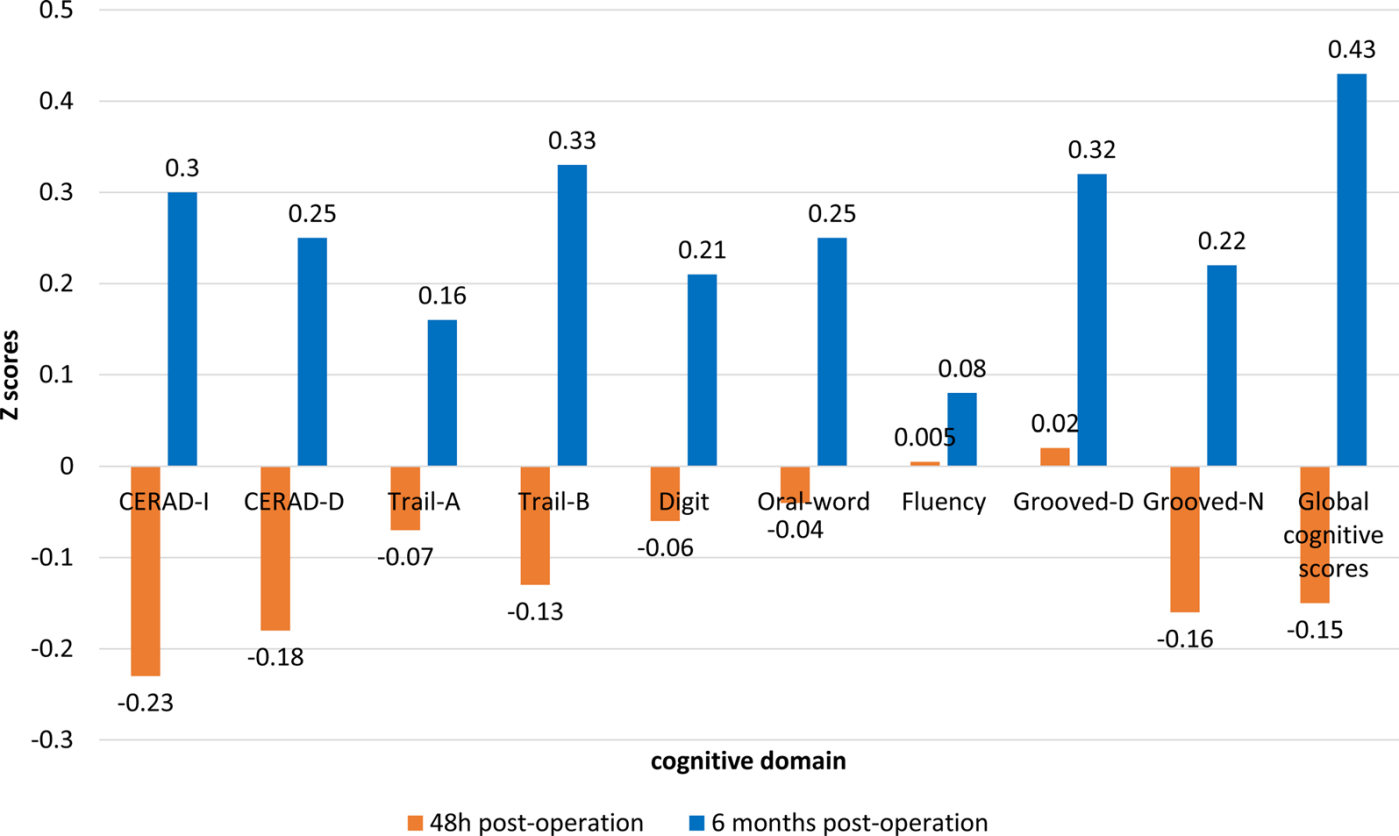
Changes in individual Z scores and global cognitive scores. The mean value of the scores of the baseline after standardization is 0. CERAD-I: CERAD Auditory-Verbal Learning Test-Immediate; CERAD-D: CERAD Auditory-Verbal Learning Test-Delayed; Trail-A: Trail Making Task Part A; Trail-B: Trail Making Task Part B; Digit: Digit Symbol Substitution Test; Oral-word: Controlled Oral Word Association Test; Fluency: CERAD Semantic Fluency Test; Grooved-D: Grooved Pegboard Test (Dominant Hand); Grooved-N: Grooved Pegboard Test (Non-dominant Hand)

172 patients in the study group accepted a 6-month follow up for an extra cognitive assessment, including 23 patients with POCD at 48 h post-ablation and 149 patients without POCD. In these 172 patients, the global cognitive scores decreased in 48 h post-operation tests (0 ± 1 vs − 0.15 ± 1.10, *P* < 0.001) and improved significantly at 6 months post-operation (0 ± 1 vs 0.43 ± 0.92, *P* < 0.001). Cognitive scores at 6 months improved significantly in memory: CERAD-I (0 ± 1 vs 0.30 ± 0.90, *P* < 0.001), CERAD-D (0 ± 1 vs 0.25 ± 0.99, *P* < 0.001); in executive function: Trail-A (0 ± 1 vs 0.16 ± 0.80, *P* < 0.001), Trail-B (0 ± 1 vs 0.33 ± 0.84, *P* < 0.001); in working memory and processing function: Digit Symbol Substitution Test (0 ± 1 vs 0.21 ± 1.01, *P* < 0.001), in verbal fluency test: Controlled Oral Word Association Test (0 ± 1 vs 0.25 ± 0.97, *P* < 0.001), and in visual-motor coordination: Grooved-D (0 ± 1 vs 0.32 ± 0.69, *P* < 0.001), Grooved-N tests (0 ± 1 vs 0.22 ± 0.69, *P* < 0.001). Detailed analysis of changes in global cognitive scores and individual tested Z scores from baseline to 6 months follow-up were shown in Fig. 2.

**Fig. 3:**
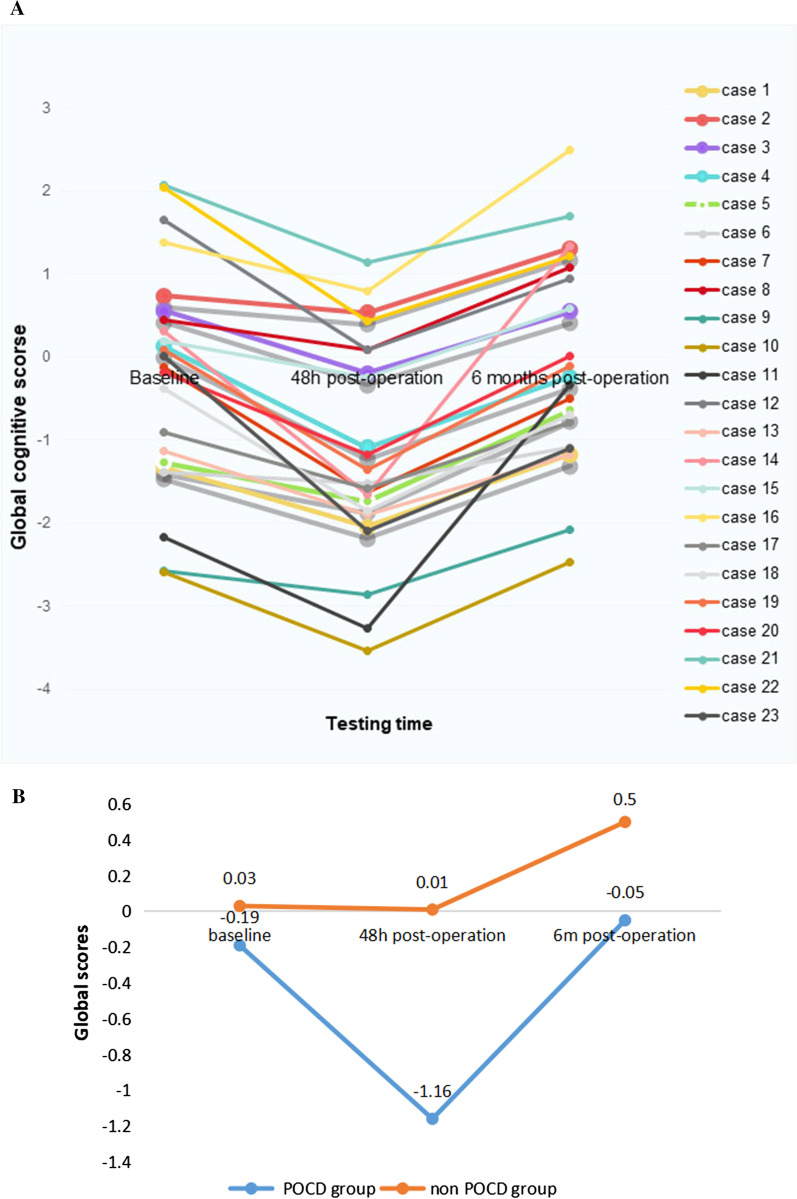
Global cognitive scores**. a** The trajectories of global cognitive scores in patients with POCD at 48 h pose-operation. **b** The comparison between the POCD group and the non POCD group at baseline, 48 h post-ablation, 6 months post-ablation

In the 23 patients with POCD at 48 h after the procedure, global cognitive performance at 6 months has no significant difference to that at baseline (− 0.05 ± 1.25 vs − 0.19 ± 1.33, *P* = 0.32), and 13 of them had higher scores than baseline level. The trajectories of global cognitive scores in 23 patients with POCD at 48 h after the procedure are shown in Fig. 3a. The comparison between the POCD group and the non POCD group at baseline, 48 h post-ablation, 6 months post-ablation were shown in Fig. 3b.

## Discussion

The main findings of this study were that 13.7% (26/190) of patients undergoing AF ablation had postoperative cognitive decline at 48 h after AF ablation, even under local anesthesia, which was associated with poor anticoagulation before and during the operation procedure. The memory and executive function were the most commonly affected cognitive domains. However, this cognitive impairment seemed to be only temporary. In overall population, AF ablation might be associated with cognition improvement after 6 months post-operation.

As previously reported, the incidence of subclinical post-procedural cognitive impairment was significantly higher at 48 h after the procedure under general anesthesia [5]. Similarity, POCD was observed in the present study but with a lower 48-h incidence (13.7%), indicating general anesthesia may affect patients’ cognitive function shortly after the operation.

Besides anesthesia, the underlying mechanism for cognitive impairment caused by ablation itself remains controversial. It has been shown that "subclinical" or "silent" strokes contribute to memory impairment and cognitive decline [21–23]. AF patients have a higher risk of silent brain lesions and cognitive impairment [24]. A previous study reported a high incidence of AF ablation-related subclinical intracranial embolic events, which was estimated at 50%, depending on the specific MRI protocols used to identify acute embolic lesions [25]. In post-AF ablation patients, adverse neuropsychological changes in patients’ verbal memory, conjoined with ischemic brain injury on DWI have been observed [9]. Accordingly, we found that the quality of peri-procedural anticoagulation seems to be crucial to POCD. On the contrary, it has also been showed that no neuropsychological effect was found from these incident brain lesions in 16 of 37 patients undergoing AF ablation [7]. However, in this study, patients’ post-operative cognitive status was only assessed 6 months after ablation, when many of brain lesions have ceased to persist. Therefore, a study with a larger sample size and more frequent cognitive monitor is expected in the future.

Generally, the AF ablation procedure might induce thromboembolic events through several potential mechanisms. First, the catheter and sheath maneuver, as well as endothelial ablation in the left atrium, are prone to thrombus formation. Second, AF ablation itself induces systemic coagulation system activation and may cause left atrial diastolic dysfunction, further increasing thromboembolism risk [26]. Third, perioperative anticoagulation interruption and bridging therapy placed atrial fibrillation patients in an unstable anticoagulation state, thus increasing the risk for brain infarction and microbleeds, which has also attributed to POCD [27, 28]. Aside from the factors mentioned above, increased age, particular disease states might also be risk factors for POCD [29].

It has also been reported that left atrial access time is associated with POCD, as it may expose patients to a higher chance of incident brain lesion [5]. Intriguingly, the minimum intraoperative ACT values less than 300 s during the ablation procedure was found to be an independent risk factor for POCD, indicating that adequate anticoagulation during the procedure might exert an effect in preventing POCD. Several studies have found that more aggressive anticoagulation during ablation were associated with a lower risk of symptomatic embolic events [30, 31] and a lower frequency of LA thrombus formation [32]. Regarding asymptomatic embolic events, studies have showed that lower intraprocedural ACT [33, 34] and increased procedure duration before systemic heparinization [35] were related with an increased risk of asymptomatic cerebral embolism (ACE), and that the presence of spontaneous echo contrast in the LA was also related with ACE risk [35, 36]. These data suggested that well-controlled intraprocedural ACT and earlier heparinization may reduce the risk of ACE, as confirmed in a prospective, multicenter study [27].

Irregular anticoagulation prior to the ablation is also a POCD risk factor, even if no thrombosis was detected by pre-procedural esophageal ultrasound. Long-term anticoagulation might decrease potential thromboses in the left atrial appendage or left atrium that cannot be detected by esophageal ultrasound. The catheter operation in the left atrium and conversion to the sinus rhythm may cause these small clots to fall off and cause an asymptomatic embolism, thereby affecting the patient's cognitive function. Routine administration of oral anticoagulants for one month before ablation eliminated most of the tiny clots and thus might have the potential to reduce POCD. Moreover, the risk of thrombosis resulting from LMWH bridging should not be neglected. Previous studies have shown a 2% incidence of silent cerebral lesions in AF ablation patients under uninterrupted warfarin anticoagulation and a 14% in those with LMWH bridging [27]. In this study, the association of LMWH bridging with POCD was not observed, which was possibly due to that the small size of uninterrupted patients (n = 27, 14.2%) is not sufficient to illustrate the difference.

Emerging evidence has found that AF ablation improves long-term cognitive function, and this improvement begins to appear in the third month after ablation [10]. In the present study, cognitive improvement was also observed at the 6-month follow up, while the trajectories of each cognitive domain were further described. Both clinically and statistically significant changes were observed in memory, executive function, working memory and processing function, verbal fluency test and visual-motor coordination, which conforms well to the cognitive domains that are most commonly impaired in AF patients [24, 37]. The recovery of sinus rhythm after AF ablation improves atrioventricular synchronization as well as the systolic and diastolic function [38], which may enhance cerebral perfusion, promoting the recovery and improvement of cognitive function.

Most POCD cases had a tendency of recovery at 6 months, consistent with a recent study, which reported that most acute brain lesions after ablation disappeared at 6 months post-ablation depending on the specific MRI [7].

Although the patients’ cognitive function was carefully measured for POCD diagnosis, diffusion-weighted MRI were not performed before and after ablation. Therefore, the estimation accuracy of the true lesion rate and the impact of embolic events on the cognitive outcome may be affected. With the recent development and widespread usage of an uninterrupted perioperative NOAC strategy, a better anticoagulation strategy before and during the ablation procedure, the perioperative stroke/TIA rate may have been lowered, thus, the results may have overestimated the POCD rate.

## Conclusions

Atrial fibrillation ablation is associated with a 13.7% prevalence of POCD within 48 h after ablation procedures. Patients, who were not on routine anticoagulants within one month prior to the ablation procedure and with a minimum intraoperative ACT value of less than 300 s, were found to have a higher association with POCD events. The POCD is temporary in most patients, and a general improvement was observed in cognitive function at 6 months after the procedure. Further studies regarding the long-term effects of catheter ablation on patients' cognitive function are warranted for a more comprehensive understanding in this area.

## Figures and Tables

**Fig. 4:**
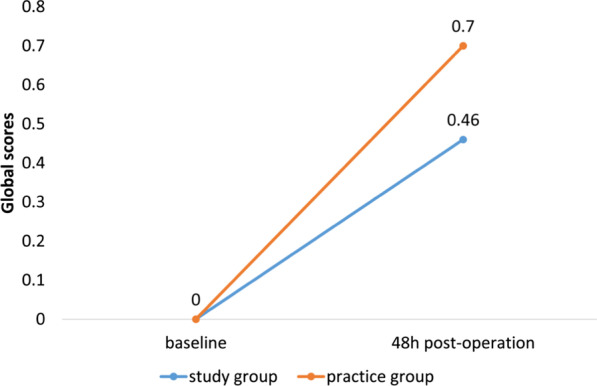
The global cognitive scores at baseline and 48 h post ablation for the study group and the practice group

## Data Availability

The datasets used and/or analyzed during the current study are available from the corresponding author on reasonable request.

## Appendix

See Table 4 and Fig. 4.

**Table 4:** Description of Neuropsychological Tests

Test	Description	Cognitive domain
CERAD Auditory-Verbal Learning Test-Immediate	First, the patients were read ten words. Then, they were required to recall the words as many as possible. This was repeated twice; yet, the word order was changed every time. The maximum number of words correctly recalled was recorded	Memory
CERAD Auditory-Verbal Learning Test-Delayed	After 15 min, the patients were required to recall as many as possible of the ten words from the CERAD Auditory-Verbal Learning Test-Immediate without rereading. The number of words correctly remembered was recorded	Memory
Trail Making Task Part A	Twenty-five circles numbered 1–25 distributed over a sheet of paper. The patients were instructed to connect the circles by drawing lines with the numbers in ascending order as fast as possible. The time spent to complete the task was the score.to be recorded	Executive functioning
Trail Making Task Part B	Twenty-five circles were distributed over a sheet of paper; the circles included either numbers (1–13) or letters (A-L). The patients were instructed to connect the circles by drawing lines between the numbers and letters in ascending order (i.e.,1-A-2-B-3-C, and so on). The time spent to complete the task was recorded as the score	Executive functioning
Digit Symbol Substitution Test	The patients were required to reproduce coded symbols on paper as many as possible within blank boxes beneath the digits randomly generated, based on a coding scheme that paired the digits with symbols. The number of correct symbols reproduced within 90 s was recorded as the score	Memory and processing function
Controlled Oral Word Association Test	The patients were instructed to say words as many as possible within 60 s that start with a given Chinese character component. “氵, 亻, and 日” were tested	Verbal fluency test
CERAD Semantic Fluency Test	The patients were instructed to make a list of words as many as possible within 60 secondsfrom a predefined category (fruits). The number of correct answers was recorded	Verbal fluency test of semantic memory
Grooved Pegboard Test (Dominant Hand)	The patients were required to insert 25 keyed pegs into a pegboard which was specially designed with randomly positioned slots. The pegs must match the hole, or they cannot be inserted.The time spent to complete the task was recorded	Requires complex visual-motor coordination and evaluates the lateralized injury
Grooved Pegboard Test (Nondominant Hand)	The patients completed the same task with the nondominant hand. The time taken to complete the task was recorded	Requires complex visual-motor coordination and evaluates the lateralized injury

Adapted from Silbert et al. [13]

CERAD , the Consortium to Establish a Registry for Alzheimer’s Disease

## Abbreviations

AF: Atrial fibrillation
RCI: Reliable change index
POCD: Postoperative cognitive dysfunction
ACT: Activated clotting time
QoL: Quality of life
RCI: Reliable change index
TEE: Transesophageal echocardiography
INR: International normalized ratio
NOAC: New oral anticoagulants
PAF: Paroxysmal atrial fibrillation
SD: Standard deviation
MMSE: Mini-Mental State Examination

## References

[1] Calkins H, Reynolds MR, Spector P (2009). Treatment of atrial fibrillation with antiarrhythmic drugs or radiofrequency ablation: two systematic literature reviews and meta-analyses. Circ Arrhythm Electrophysiol.

[2] Morillo CA, Verma A, Connolly SJ (2014). Radiofrequency ablation vs antiarrhythmic drugs as first-line treatment of paroxysmal atrial fibrillation (RAAFT-2): a randomized trial. JAMA.

[3] Wazni OM, Marrouche NF, Martin DO (2005). Radiofrequency ablation vs antiarrhythmic drugs as first-line treatment of symptomatic atrial fibrillation: a randomized trial. JAMA.

[4] Conen D, Rodondi N, Muller A (2019). Relationships of overt and silent brain lesions with cognitive function in patients with atrial fibrillation. J Am Coll Cardiol.

[5] Medi C, Evered L, Silbert B (2013). Subtle post-procedural cognitive dysfunction after atrial fibrillation ablation. J Am Coll Cardiol.

[6] Haeusler KG, Koch L, Herm J (2013). 3 Tesla MRI-detected brain lesions after pulmonary vein isolation for atrial fibrillation: results of the MACPAF study. J Cardiovasc Electrophysiol.

[7] Herm J, Fiebach JB, Koch L (2013). Neuropsychological effects of MRI-detected brain lesions after left atrial catheter ablation for atrial fibrillation: long-term results of the MACPAF study. Circ Arrhythm Electrophysiol.

[8] Von Bary C, Deneke T, Arentz T (2015). Silent cerebral events as a result of left atrial catheter ablation do not cause neuropsychological sequelae–a MRI-controlled multicenter study. J Interv Card Electrophysiol.

[9] Schwarz N, Kuniss M, Nedelmann M (2010). Neuropsychological decline after catheter ablation of atrial fibrillation. Heart Rhythm.

[10] Jin MN, Kim TH, Kang KW (2019). Response by Jin et al to Letter Regarding Article, "Atrial fibrillation catheter ablation improves 1-year follow-up cognitive function, especially in patients with impaired cognitive function". Circ Arrhythm Electrophysiol.

[11] Canadian study of health and aging: study methods and prevalence of dementia. Cmaj, 1994, 150(6): 899–913. PMC1486712 8131123

[12] Evered LA, Silbert BS, Scott DA (2009). Plasma amyloid beta42 and amyloid beta40 levels are associated with early cognitive dysfunction after cardiac surgery. Ann Thorac Surg.

[13] Silbert BS, Scott DA, Evered LA (2006). A comparison of the effect of high- and low-dose fentanyl on the incidence of postoperative cognitive dysfunction after coronary artery bypass surgery in the elderly. Anesthesiology.

[14] Evered LA, Silbert BS, Scott DA (2010). Postoperative cognitive dysfunction and aortic atheroma. Ann Thorac Surg.

[15] Evered LA, Silbert BS, Scott DA (2011). Preexisting cognitive impairment and mild cognitive impairment in subjects presenting for total hip joint replacement. Anesthesiology.

[16] Rasmussen LS, Larsen K, Houx P (2001). The assessment of postoperative cognitive function. Acta Anaesthesiol Scand.

[17] Singh-Manoux A, Fayosse A, Sabia S (2017). Atrial fibrillation as a risk factor for cognitive decline and dementia. Eur Heart J.

[18] Medi C, Sparks PB, Morton JB (2011). Pulmonary vein antral isolation for paroxysmal atrial fibrillation: results from long-term follow-up. J Cardiovasc Electrophysiol.

[19] Rosso R, Sparks PB, Morton JB (2010). Vagal paroxysmal atrial fibrillation: prevalence and ablation outcome in patients without structural heart disease. J Cardiovasc Electrophysiol.

[20] Dong JZ, Sang CH, Yu RH (2015). Prospective randomized comparison between a fixed '2C3L' approach vs. stepwise approach for catheter ablation of persistent atrial fibrillation. Europace.

[21] Vermeer SE, Longstreth WT, Koudstaal PJ (2007). Silent brain infarcts: a systematic review. Lancet Neurol.

[22] Atanassova PA, Massaldjieva RI, Dimitrov BD (2016). Early neurological and cognitive impairments in subclinical cerebrovascular disease. Neurol India.

[23] Blum S, Luchsinger JA, Manly JJ (2012). Memory after silent stroke: hippocampus and infarcts both matter. Neurology.

[24] Gaita F, Corsinovi L, Anselmino M (2013). Prevalence of silent cerebral ischemia in paroxysmal and persistent atrial fibrillation and correlation with cognitive function. J Am Coll Cardiol.

[25] Deneke T, Jais P, Scaglione M (2015). Silent cerebral events/lesions related to atrial fibrillation ablation: a clinical review. J Cardiovasc Electrophysiol.

[26] Haeusler KG, Kirchhof P, Endres M (2012). Left atrial catheter ablation and ischemic stroke. Stroke.

[27] Di Biase L, Gaita F, Toso E (2014). Does periprocedural anticoagulation management of atrial fibrillation affect the prevalence of silent thromboembolic lesion detected by diffusion cerebral magnetic resonance imaging in patients undergoing radiofrequency atrial fibrillation ablation with open irrigated catheters? Results from a prospective multicenter study. Heart Rhythm.

[28] Zhao Y, Yang Y, Tang X (2017). New oral anticoagulants compared to warfarin for perioperative anticoagulation in patients undergoing atrial fibrillation catheter ablation: a meta-analysis of continuous or interrupted new oral anticoagulants during ablation compared to interrupted or continuous warfarin. J Interv Card Electrophysiol.

[29] Kotekar N, Shenkar A, Nagaraj R (2018). Postoperative cognitive dysfunction - current preventive strategies. Clin Interv Aging.

[30] Di Biase L, Burkhardt JD, Mohanty P (2010). Periprocedural stroke and management of major bleeding complications in patients undergoing catheter ablation of atrial fibrillation: the impact of periprocedural therapeutic international normalized ratio. Circulation.

[31] Wazni OM, Rossillo A, Marrouche NF (2005). Embolic events and char formation during pulmonary vein isolation in patients with atrial fibrillation: impact of different anticoagulation regimens and importance of intracardiac echo imaging. J Cardiovasc Electrophysiol.

[32] Ren JF, Marchlinski FE, Callans DJ (2005). Increased intensity of anticoagulation may reduce risk of thrombus during atrial fibrillation ablation procedures in patients with spontaneous echo contrast. J Cardiovasc Electrophysiol.

[33] Gaita F, Caponi D, Pianelli M (2010). Radiofrequency catheter ablation of atrial fibrillation: a cause of silent thromboembolism? Magnetic resonance imaging assessment of cerebral thromboembolism in patients undergoing ablation of atrial fibrillation. Circulation.

[34] Scaglione M, Blandino A, Raimondo C (2012). Impact of ablation catheter irrigation design on silent cerebral embolism after radiofrequency catheter ablation of atrial fibrillation: results from a pilot study. J Cardiovasc Electrophysiol.

[35] Sakamoto T, Kumagai K, Nishiuchi S (2013). Predictors of asymptomatic cerebral infarction associated with radiofrequency catheter ablation for atrial fibrillation using an irrigated-tip catheter. Europace.

[36] Martinek M, Sigmund E, Lemes C (2013). Asymptomatic cerebral lesions during pulmonary vein isolation under uninterrupted oral anticoagulation. Europace.

[37] Nishtala A, Piers RJ, Himali JJ (2018). Atrial fibrillation and cognitive decline in the Framingham Heart Study. Heart Rhythm.

[38] Cha YM, Wokhlu A, Asirvatham SJ (2011). Success of ablation for atrial fibrillation in isolated left ventricular diastolic dysfunction: a comparison to systolic dysfunction and normal ventricular function. Circ Arrhythm Electrophysiol.

